# Interobserver Variability of Target Volumes Delineated in the Supine and Prone Positions Based on Computed Tomography Images for External-Beam Partial Breast Irradiation After Breast-Conserving Surgery: A Comparative Study

**DOI:** 10.3389/fonc.2020.00323

**Published:** 2020-04-08

**Authors:** Ting Yu, YanKang Li, Wei Wang, Fengxiang Li, Jinzhi Wang, Min Xu, Yingjie Zhang, Jianbin Li, Jinming Yu

**Affiliations:** ^1^Key Laboratory of Cancer Prevention and Therapy, National Clinical Research Center for Cancer, Tianjin's Clinical Research Center for Cancer, Tianjin Medical University Cancer Institute and Hospital, Tianjin Medical University, Tianjin, China; ^2^Department of Radiation Oncology, Shandong Cancer Hospital and Institute (Shandong Cancer Hospital), Shandong First Medical University and Shandong Academy of Medical Sciences, Jinan, China; ^3^Cheeloo College of Medicine, Shandong University, Jinan, China

**Keywords:** external-beam partial breast irradiation, target volume delineation, interobserver variability, supine position, prone position

## Abstract

**Background:** Although the supine position remains the dominant position for external-beam partial breast irradiation (EB-PBI), the advantages of administering EB-PBI in the prone position have been recognized. The interobserver variability between target volumes delineated in the different positions for EB-PBI after breast-conserving surgery needs to be investigated.

**Methods:** Twenty-seven patients suitable for EB-PBI were enrolled from July 2016 to April 2017. Supine and prone simulation CT images were sequentially acquired for all enrolled patients during free breathing. Five experienced radiotherapists delineated the target volumes for all patients on supine and prone simulation CT images. The selected parameters, including target volumes, the coefficient of variation (COV), the matching degree (MD), and so on, were calculated to analyze the interobserver variability.

**Results:** Regardless of the patient position, the interobserver variability between tumor bed (TB) and clinical target volume (CTV) measurements in supine and prone positions were statistically significant (*F* = 31.34, 19.467; 44.000, 41.985; *P* = 0.000, 0.001; 0.000, 0.001). The interobserver variability of COV_CTV_ was significantly greater in the supine position than in the prone position (*T* = 2.64, *P* = 0.014). Furthermore, the interobserver variabilities of MD_TB_ and MD_CTV_ were statistically lower in the supine position than in the prone position (*Z* = −3.460, −3.195, *P* = 0.000, 0.001).

**Conclusion:** When delineating the target volume for EB-PBI, the interobserver variability in the prone position was lower than that in the supine position. Hence, the administration of EB-PBI in the prone position during free breathing is a reasonable option.

## Introduction

Recently, partial breast irradiation (PBI) has been considered an alternative to whole breast irradiation (WBI) for patients with early-stage breast cancer after breast-conserving therapy (1–3). Previous results from the UK IMPORT LOW trial (4) showed the non-inferiority of PBI compared with standard WBI in terms of breast cancer outcomes and that PBI had equivalent or fewer late normal-tissue adverse effects than WBI. Furthermore, external-beam partial breast irradiation (EB-PBI) is one of the main approaches for PBI. The accuracy of target volume delineation is greatly important in EB-PBI. Inadequate definitions of the gross tumor volume (GTV) or clinical target volume (CTV) can lead to systematic geometric errors or radiation errors to the tumor in individual patients during radiation therapy (5), further affecting the organs at risk (OARs), influencing the long-term survival of patients and increasing the risk of local recurrence.

In fact, target definition for postoperative EB-PBI is a complicated process. The definition of the tumor bed (TB) is affected by several factors, such as the operator, surgical method, operation cavity, interobserver variability, and delineation guidelines (6). One of the potential factors that influences the radiotherapy errors observed in target delineation is interobserver variability (7). Previous studies (8, 9) have shown that interobserver variability widely depends on the delineation guidelines, the observers, seroma clarity, surgical clips, and other aspects. Moreover, several studies (10–15) have shown that the interobserver variability in delineating target volumes for breast cancer radiotherapy was significant. Landis et al. (16) also confirmed that when the cavity visualization score (CVS) was 4, the average percent overlap between the planning target volume (PTV) delineated by two observers only reached up to 77%. In addition, a multi-institutional study from the radiation therapy oncology group (RTOG) further indicated that the differences between target volumes and OARs delineated by observers for breast irradiation appear to be clinically and dosimetrically significant (17).

However, the current studies on interobserver variability in breast cancer irradiation are almost based on the supine position. Although the supine position is still the most common approach for adjuvant radiotherapy after breast-conserving surgery (BCS), the incidence of pulmonary and cardiovascular events increases over time after radiotherapy administered in the supine position (18, 19). Moreover, as the application of radiotherapy in the prone position has increased, the advantages of the prone position in reducing radiation exposure to the lung and improving dose homogeneity have been observed to be more significant than those in the supine position (20–22). Additionally, placing patients in the prone position during WBI can also reduce the large dose distribution in the target (23, 24). Since then, the prone breast irradiation has gained much attention. However, whether the interobserver variability in delineating target volumes based on prone CT will improve has not been clearly established. Therefore, this study aims to investigate the interobserver and intraobserver variability for EB-PBI on supine and prone simulation CT scans after BCS.

## Methods

### Patient Selection

Breast cancer patients eligible for EB-PBI after BCS were recruited in this study. All selected patients underwent lumpectomy, and patients who underwent oncoplastic BCS were excluded. At least five clips were positioned at the central bottom and lateral edges of the surgical cavity to mark the TB boundaries. All patients underwent axial 3DCT simulation scanning successively during free breathing in the supine and prone positions. In addition, the enrolled patients had a seroma clarity score of <3. The research was performed in accordance with relevant regulations, and all patients in this study provided written informed consent. The study was authorized by the Institutional Research Ethics Board of the Shandong Tumor Hospital Ethics Committee.

### Simulation Scanning and Image Acquisition

All 3DCT data sets were acquired with a thickness of 3 mm on a 16-slice CT scanner (Philips Brilliance Bores CT, Netherlands) during free breathing. For the supine position, the patients were immobilized in the treatment position on an inclined breast board with both arms raised above the head to expose adequately the breast. Afterward, the patients were fixed on a prone dedicated treatment board (CIVCO HorizonTM Prone Breast Bracket–MTHPBB01) with no inclination degree. And the arms were in abduction, with the hands above the head. Due to the open aperture on one side of the simulation board, the ipsilateral breast could hang freely away from the chest wall. All CT images were transferred to MIM vista version 6.7.6 (MIM Software; Cleveland, OH) software.

### Target Volume Delineation

All target volumes were delineated by five radiotherapists with extensive experience in delineating breast target volumes. The TB was delineated based only on the surgical clips, and the TBs delineated based on the supine and prone CT images were defined as TBs and TBp, respectively. The CTV comprised of the TB plus a 10-mm margin and was defined as CTVs and CTVp for the supine and prone positions, respectively. The anterior border of the CTV was limited to being 5 mm from the skin surface, and the posterior border did not exceed the gland-pectorales interface. Furthermore, one of the five radiotherapists contoured the TB based only on the surgical clips three times on the supine and prone CT images of each patient. The delineation criteria were consistent in both the supine and prone setups.

### Parameter Evaluation

To quantify the interobserver variability, the target volumes, including the TB and the CTV, were calculated separately in the different positions. The distance between the center of the TB and the chest wall (D_T−C_) was measured in both the prone and supine positions.

The interobserver coefficient of variation in each position (COVs and COVp) was calculated for each patient and defined as the ratio between the standard deviation and the average of the target volumes. The matching degree (MD) was computed according to the methods by Geets (25). For each patient, the ratio between the intersection volume (the intersection among the volumes delineated by the five observers) and the union volume (the union among the volumes delineated by the five observers) was calculated in each position and termed MDs and MDp, respectively. The variability of the volumes and above parameters based on the same observer was also calculated.

### Statistical Analyses

The SPSS 19.0 software (IBM Corporation, Armonk, NY, USA) was used for the statistical analysis. Data that did not follow a normal distribution were analyzed by the Wilcoxon signed-rank test and described using medians and ranges. Data that followed a normal distribution were analyzed by paired-samples *t*-tests and described using means and standard deviations. To investigate the interobserver variability in the delineating target volumes on supine and prone simulation 3DCTs, a univariate analysis of variance (ANOVA) was used to compare the differences in the target volumes, COVs (COVs and COVp), and MDs (MDs and MDp) between observers. The relevance between target volumes was established by the Spearman rank correlation analysis. Data were considered statistically significant at *P* < 0.05.

## Results

### Patient Characteristics

A total of 27 early-stage breast cancer patients after BCS were enrolled in this study from July 2016 to April 2017. The patients, with a median age of 41 years (range, 28–69 years), had a clinical T1N0M0 staged adenocarcinoma of the breast. The average volume of the breasts was 644.93 cm^3^. In addition, patients underwent lumpectomy with sentinel lymph node dissection (SLND) or axillary lymph node dissection (ALND), and the pathological reports after BCS had showed tumor-negative surgical margins. The patient and tumor characteristics are listed in Table 1.

**Table 1:** Patient and tumor characteristics.

**Variable**	**Value**
Age, years	
Median	41
Range	28–69
Tumor size	
≥10 <20 mm	11
≥20 mm	16
Breast side	
Left	15
Right	12
Localization of the tumor bed	
UOQ	17
LOQ	2
Central portion of the breast	1
UIQ	6
LIQ	1
Tumor characteristics	
Ductal carcinoma *in situ*	4
Invasive ductal carcinoma	20
Invasive lobular carcinoma	0
Cribriform carcinoma	1
Mucinous carcinoma	2

*UOQ, upper outer quadrant; LOQ, lower outer quadrant; UIQ, upper inner quadrant; LIQ, lower inner quadrant*.

### Comparison of the Target Volumes Delineated by Different Observers

The TBs, TBp, CTVs, and CTVp volumes are listed in Tables 2, 3. Regardless of the patient position, the interobserver variabilities for TB and CTV measurements delineated in the supine and prone positions were both statistically significant (*F* = 31.34, 19.467; 44.000, 41.985; *P* = 0.000, 0.001; 0.000, 0.001). However, the variabilities among the TBs and TBp and CTVs and CTVp measurements delineated by each observer were not significantly different (all *P* > 0.05). The intersections of TB areas delineated by the five radiotherapists were 7.67 and 8.25 cm^3^ in the supine and prone positions, respectively, which showed no significant difference (*T* = −1.506, *P* = 0.144). However, the intersections of CTV were 57.43 and 51.64 cm^3^, respectively (*T* = −2.735, *P* = 0.011); therefore, the CTV intersection in the prone position was 5.79 cm^3^ greater than that in the supine position. Regardless of the patient position, the ANOVA test showed no significant differences in the volume variability among the TBs repeatedly delineated three times by the same observer in the supine and prone positions (*F* = 1.556, 2.667, *P* = 0.459, 0.264). Moreover, no significant differences in the volume variability among the TB areas delineated by the same observer were evident between the supine and prone positions (*Z* = −1.946, −1.730, −1.922, *P* = 0.052, 0.084, 0.055).

**Table 2:** Comparison of the TBs delineated in the supine and prone positions by five observers (cm^3^).

**Observer**	**TBs**	**TBp**	***Z***	***P*-value**
1	11.12 (40.91–5.12)	10.28 (29.71–3.33)	−1.490	0.136
2	14.60 (40.00–3.66)	14.68 (51.41–3.70)	−0.709	0.478
3	17.39 (36.24–6.60)	14.70 (53.59–7.33)	−1.201	0.23
4	14.81 (34.50–8.27)	15.25 (30.58–5.41)	−1.417	0.156
5	16.74 (33.60–8.51)	16.32 (45.72–7.13)	−0.961	0.337
*F*	31.340	44.000		
*P*-value	0.000	0.000		

*TBs, tumor bed delineated in the supine position based on the surgical clips; TBp, tumor bed delineated in the prone position based on the surgical clips*.

**Table 3:** Comparison of the CTV measurements delineated in the supine and prone positions by five observers (cm^3^).

**Observer**	**CTVs**	**CTVp**	***Z***	***P*-value**
1	61.52 (156.84–30.76)	58.25 (145.16–27.62)	−1.153	0.249
2	67.52 (152.46–33.05)	72.19 (169.14–33.37)	−1.033	0.302
3	66.39 (129.33–43.76)	69.84 (186.13–39.80)	−1.009	0.313
4	70.90 (122.84–39.57)	71.01 (137.35–34.57)	−0.961	0.337
5	68.51 (127.56–42.39)	72.84 (151.56–40.04)	−0.745	0.456
*F*	19.467	41.985		
*P*-value	0.001	0.000		

*CTVs, clinical target volume delineated in the supine position; CTVp, clinical target volume delineated in the prone position*.

### Evaluation of the Target Volume Parameters Among Observers

The interobserver variability of COV_TB_ was not statistically significant between the supine and prone positions (*Z* = 1.236, *P* = 0.228), but unlike COV_TB_, the COV_CTV_ increased significantly in the supine position compared with that in the prone position (*T* = 2.64, *P* = 0.014, Table 4). Table 5 lists the differences in MD between the volumes delineated in the supine and prone positions. The MD_TB_ and MD_CTV_ were both significantly smaller in the supine position than in the prone position (*T* = −4.497, *P* = 0.000; *Z* = −3.195, *P* = 0.001). Moreover, a statistically significant inverse correlation was found between COV_CTV_ and MD_CTV_ (*r* = −0.772, −0.857; *P* = 0.000, 0.000). However, similar to the intraobserver variability of COV and MD, no significant differences were observed between the supine and prone positions (*Z* = −1.201, −0.721; *P* = 0.230, 0.471).

**Table 4:** Evaluation of the interobserver variability parameters for COV.

**Parameter**	**TB**	**CTV**
COVs	0.26 ± 0.08	0.14 ± 0.06
COVp	0.25 ± 0.07	0.11 ± 0.04
*T*	1.236	2.64
*P*-value	0.228	0.014

*COVs, the interobserver coefficient of variation in the supine position; COVp, the interobserver coefficient of variation in the prone position*.

**Table 5:** Evaluation of the interobserver variability parameters for MD.

**Parameter**	**TB**	**CTV**
MDs	0.28 (0.53–0.15)	0.54 (0.75–0.36)
MDp	0.33 (0.51–0.18)	0.61 (0.73–0.27)
*Z*	−3.46	−3.195
*P*-value	0.001	0.001

*MDs, the matching degree in the supine position; MDp, the matching degree in the prone position*.

### Comparison of the D_T-C_ Among Observers

Regardless of the patient position, the interobserver variabilities of D_T−C_ in both the supine and prone positions were statistically significant (*F* = 37.847, 13.067; *P* = 0.000, 0.011), and the D_T−C_ measurements defined by each observer were all significantly longer in the prone position than in the supine position (all *P* < 0.05, Table 6). In our study, the D_T−C_ values were well-correlated with the volume of the ipsilateral breast in both the supine and prone positions (*r* = 0.716, 0.752, 0.696, 0.783, 0.695; 0.761, 0.732, 0.723, 0.785, 0.765, all *P* < 0.05). Moreover, a statistically significant inverse correlation was found between the D_T−C_ and the ratio of the CTV to the volume of the treated breast in the supine and prone positions (*r* = −0.621, −0.484, −0.487, −0.568, −0.474; −0.426, −0.471, −0.424, −0.391, −0.457, all *P* < 0.05). Regardless of the patient position, no significant differences were observed in the D_T−C_ measurements delineated three times by the same observer in the supine and prone positions (*F* = 1.086, 0.980; *P* = 0.581, 0.613). However, the D_T−C_ measurements delineated by the same observer were relatively lower in the supine position than those in the prone position (*Z* = −4.121, −3.797, −3.820, all *P* < 0.05).

**Table 6:** Comparison of the D_T−C_ measurements among observers in the supine and prone positions (cm).

**Observer**	**D_**T-Cs**_**	**D_**T-Cp**_**	***Z***	***P*-value**
1	0.57 (1.92–0.09)	1.02 (6.99–0.21)	−4.121	0.000
2	0.67 (2.21–0.14)	0.97 (6.62–0.16)	−4.037	0.000
3	0.47 (2.03–0.08)	0.91 (6.60–0.17)	−4.001	0.000
4	0.66 (1.95–0.08)	0.91 (6.60–0.17)	−3.965	0.000
5	0.76 (2.24–0.16)	1.02 (6.78–0.31)	−3.724	0.000
*F*	37.847	13.067		
*P*-value	0.000	0.011		

*D_T−Cs_, the distance between the center of the TB and the chest wall in the supine position; D_T−Cp_, the distance between the center of the TB and the chest wall in the prone position*.

## Discussion

Our study demonstrated that for both supine and prone EB-PBI, large variability in the delineation of the TB and CTV measurements can exist among breast cancer radiation oncologists. At present, a few studies have focused on the interobserver variability of target volumes delineated for prone EB-PBI. However, as for supine EB-PBI, Landis et al. (16) confirmed that even among breast cancer radiation oncologists, significant differences in the delineation of TB and PTV can still be observed. Petersen et al. (10) also reported that the mean conformity index of TB areas delineated based on seroma only reached up to 0.61 among three observers, who each contoured 30 partial breast volumes. Moreover, Guo et al. (26) further clarified that for supine EB-PBI, the interobserver variability was observed between the TBs delineated based on surgical clips and those delineated based on both the clips and seroma. These observations were similar to our results. Notably, when the TB areas were repeatedly contoured by the same observer three times in our study, no significant differences were found. Therefore, the results of our study suggest that regardless of the patient position, the interobserver variability in delineating target volumes exists objectively for EB-PBI, which might be explained by the interobserver differences in recognizing the borders of the surgical clips. Moreover, highly dense glandular tissue and benign calcifications in the breast might easily be mistaken for surgical clips, which increases the difficulty of delineating targets and increases the interobserver variability.

Our study also concluded that no significant volumetric differences were observed between the TB areas delineated by each radiotherapist in the supine and prone positions, which was consistent with previous findings (27). In addition, in our study, no statistically significant differences were observed between the CTV measurements contoured by each observer between different positions, even with the help of the same delineation guidelines. This finding might be due to the CTV delineation criteria, since the CTV comprised of the TB plus a 10-mm margin; another reason could be because the breasts of Chinese women are usually small and dense. To the best of our knowledge, the enrolled patients with breast volumes more than 750 cm^3^ consisted 25% of all patients in our study. Therefore, even though the ipsilateral breast sagged in the prone position, the breast was only mildly deformed in comparison to the breast in the supine position. In other words, for patients with small breasts who underwent radiotherapy, the breast volume discrepancy may not lead to obvious morphological changes between the supine and prone positions after BCS. Furthermore, surgical clips are not always consistent with the boundary of the lumpectomy cavity (28, 29). If the surgical clips are located close to the skin or the chest wall, the CTV would be further limited from the skin surface and gland-pectorale interface. In fact, the study by Tie et al. (30) verified a similar finding.

The study by Pogson et al. (31) compared the differences in MRI and CT for breast target volume delineation in the prone and supine positions, and no clinically significant differences were observed in the volume overlap index (VOI) of the seroma between prone and supine WBI, which were 0.57 and 0.56, respectively; however, in terms of the VOI of the whole breast CTV, the prone datasets had slightly higher interobserver conformity than the supine datasets (*P* < 0.001). However, our results concluded that the MD_TB_ and MD_CTV_ were both smaller on the supine datasets than on the prone datasets, and the difference was statistically significant. Many factors may explain the above differences, such as the TB delineation criteria. Another series that studied the role of placing surgical clips at the four cardinal points of the cavity after lumpectomy in target delineation reported that the placement of the surgical clips effectively improved the accuracy of delineating the cavity for accelerated partial breast irradiation (APBI) (32). In our study, all enrolled patients had a seroma clarity score <3, and we delineated the TB based on only the surgical clips. In addition, several studies have verified that the prone position has a significant advantage over the supine position in the conformity index of the targets (27, 33). On the other hand, our results further indicated that compared with the supine position, the prone position led to a greater CTV intersection (greater by 5.79 cm^3^). Hence, for Chinese patients with early-stage breast cancer, prone EB-PBI is feasible and could minimize the interobserver variability in target delineation and effectively improve the consistency between targets delineated by different observers.

In fact, several studies have confirmed that the doses in the WBI plan, including the mean dose and the volumes that received ≥20 Gy, were significantly reduced in the prone position for the ipsilateral lung (34, 35). Verhoeven et al. (36) also reported that the minimum distance between the seroma cavity PTV and the chest wall was 7.6 mm longer in prone WBI relative to supine WBI. Kirby et al. (37) found similar results that the prone position could reduce the mean lung doses for PBI, further proving that these benefits are applied to women regardless of breast volume. Moreover, in our previous study, the D_T−C_ was relatively larger in the prone position than in the supine position, and a significant inverse correlation was observed between the D_T−C_ and the dose parameters of the lung in the prone position (27). The main reason for these differences might be because in the prone position, the ipsilateral breast moves away from the chest wall due to gravity, which increases the D_T−C_ and reduces the segmented fields through the lung, thereby protecting the lung. To the best of our knowledge, our study on the interobserver variability between target volumes delineated in the prone and supine positions is the first to address this topic using EB-PBI. We showed that even though the interobserver variability of D_T−C_ was statistically significant in both the supine and prone positions, the D_T−C_ measurements defined by each observer were all significantly longer in the prone position than in the supine position. As a consequence, prone EB-PBI has more advantages in protecting the lung than in the supine position, and this advantage is independent of the observers.

## Conclusion

Overall, interobserver volume differences could be observed when delineating target volumes for EB-PBI based on the supine or prone position. Applying prone EB-PBI is necessary to minimize the interobserver variability in target delineation and effectively improve the consistency between targets delineated by different observers. Hence, from the perspective of reducing the interobserver variability, administering EB-PBI during free breathing in the prone position is more feasible than that in the supine position.

## Data Availability Statement

The datasets analyzed in this article are not publicly available. Requests to access the datasets should be directed to lijianbin@msn.com.

## Ethics Statement

The research was performed in accordance with relevant regulations, and all patients in this study provided written informed consent. The study was approved by the Institutional Research Ethics Board of the Shandong Tumor Hospital Ethics Committee.

## Author Contributions

TY and YL contributed to patient enrollment, statistical analysis, data analysis, and manuscript writing. JL and JY participated in the study design. WW, FL, JW, MX, and YZ delineated the target volumes.

### Conflict of Interest

The authors declare that the research was conducted in the absence of any commercial or financial relationships that could be construed as a potential conflict of interest.

## Abbreviations

EB-PBI: external-beam partial breast irradiation
3DCT: three-dimensional computed tomography
BCS: breast-conserving surgery
3DCRT: three-dimensional conformal radiation therapy
TB: tumor bed
CTV: clinical target volume
COV: coefficient of variation
MD: matching degree
PBI: partial breast irradiation
WBI: whole breast radiation
OARs: organs at risk
CVS: cavity visualization score
PTV: planning target volume
*D*_T−C_: the distance between the center of the TB and the chest wall
SLND: sentinel lymph node dissection
ALND: axillary lymph node dissection
VOI: volume overlap index
UOQ: upper outer quadrant
LOQ: lower outer quadrant
UIQ: upper inner quadrant
LIQ: lower inner quadrant.
